# Limited Impact of Cannabidiol on Health-related Quality of Life of People With Long-term Controlled HIV: A Double-blind, Randomized, Controlled Trial

**DOI:** 10.1093/ofid/ofae492

**Published:** 2024-08-27

**Authors:** Tangui Barré, Clémence Couton, Abbas Mourad, Patrizia Carrieri, Camelia Protopopescu, Hélène Klein, Barbara de Dieuleveult, Laurent Hocqueloux, Lucile Mollet, Thierry Prazuck

**Affiliations:** Aix Marseille Université, Inserm, IRD, SESSTIM, Sciences Economiques & Sociales de la Santé & Traitement de l’Information Médicale, ISSPAM, Marseille, France; Service des Maladies Infectieuses et Tropicales, Centre Hospitalier Universitaire d’Orléans, Orléans, France; CBM - Centre de Biophysique Moléculaire, Orléans, France; Aix Marseille Université, Inserm, IRD, SESSTIM, Sciences Economiques & Sociales de la Santé & Traitement de l’Information Médicale, ISSPAM, Marseille, France; Aix Marseille Université, Inserm, IRD, SESSTIM, Sciences Economiques & Sociales de la Santé & Traitement de l’Information Médicale, ISSPAM, Marseille, France; Aix Marseille Université, Inserm, IRD, SESSTIM, Sciences Economiques & Sociales de la Santé & Traitement de l’Information Médicale, ISSPAM, Marseille, France; Little Green Pharma, West Perth, Western Australia, Australia; Service des Maladies Infectieuses et Tropicales, Centre Hospitalier Universitaire d’Orléans, Orléans, France; Service des Maladies Infectieuses et Tropicales, Centre Hospitalier Universitaire d’Orléans, Orléans, France; IPIC, Laboratoire Interdisciplinaire pour l’Innovation et la Recherche en Santé d’Orléans (LI²RSO), Université d’Orléans, Orléans, France; CBM - Centre de Biophysique Moléculaire, Orléans, France; Université d’Orléans, Orléans, France; Service des Maladies Infectieuses et Tropicales, Centre Hospitalier Universitaire d’Orléans, Orléans, France; IPIC, Laboratoire Interdisciplinaire pour l’Innovation et la Recherche en Santé d’Orléans (LI²RSO), Université d’Orléans, Orléans, France

**Keywords:** cannabidiol, France, HIV, quality of life, randomized controlled trial

## Abstract

**Background:**

People with HIV (PWH) with undetectable HIV viral load still have an impaired health-related quality of life (HRQoL). Cannabidiol (CBD) is a nonintoxicating cannabis-derived cannabinoid that holds promise for the treatment of many ailments. In the present study, we tested whether oral CBD-rich medication could significantly improve PWH's HRQoL.

**Methods:**

Eighty participants with undetectable HIV viral load were randomized to either a placebo or full-spectrum CBD (1 mg/kg twice a day) arm for 12 weeks plus a 4-week follow-up period. HRQoL was assessed at baseline, week 12, and week 16 using the 36-Item Short Form Health Survey questionnaire (SF-36). Primary outcomes were physical and mental component summary scores; secondary outcomes were the 8 SF-36 subscale scores. Treatment effects on outcomes were estimated using generalized estimating equations.

**Results:**

We found no effect of CBD intake on the summary score for either component. However, CBD intake was associated with a higher physical functioning score at week 12 only (regression coefficient [95% confidence interval], 7.72 [0.55–14.89]; *P* = .035). No significant main effect of CBD intake on the other HRQoL subscale scores was observed. Furthermore, there was no difference in self-reported adverse effects between the 2 arms.

**Conclusions:**

Twice-daily CBD full-spectrum oil at 1 mg/kg had no major effect on virologically suppressed PWH's HRQoL but had a positive effect on physical functioning. Further randomized controlled trials including PWH with lower baseline HRQoL are needed to confirm this finding.

## Teaser

We tested whether oral cannabidiol-rich medication could significantly improve health-related quality of life of people living with HIV. In this randomized trial, we found no treatment effect on primary outcomes.

Monitoring and improving the health-related quality of life (HRQoL) of people with HIV (PWH) is a major challenge [1]. PWH with an undetectable HIV viral load generally have significantly poorer HRQoL than the general population and people with other chronic diseases [2, 3]. Factors negatively affecting their HRQoL include low CD4 count, multimorbidity (including psychological conditions), low socioeconomic status, social isolation, stigma, and substance use [3–10].

Cannabis use is very common among PWH [11–14] and they frequently report therapeutic motivations for its use [15–18]. However, it has also been shown that the boundary between recreational and medicinal use of cannabis is porous, and that these motivations coexist among PWH users, just as for the general population [19, 20]. The beneficial effects of cannabis on the management of HIV infection and its symptoms, as well as symptoms associated with treatments—notably on vomiting, nausea, pain, appetite, weight loss, low mood, or poor sleep quality—have been widely reported [21, 22]. Moreover, anti-inflammatory effects of cannabis have also been highlighted in PWH [23–25].

Cannabidiol (CBD) is 1 of the 2 most abundant active compounds in the *Cannabis sativa* plant. A nonintoxicant, it holds promise for the treatment of many ailments. Specifically, CBD may help in the management of anxiety [26], depression [27], and sleep disorders [28], although robust and consensual unanimous data are lacking. These 3 conditions are highly prevalent in PWH [29–32]. CBD may also help to treat pain [33, 34], another condition common in PWH [35]. Again, there is not currently enough evidence to recommend it for pain treatment [33, 34]. Moreover, through its anti-inflammatory [36] and pro-intestinal integrity properties [37, 38], it is possible that CBD could lower HIV-related chronic inflammation [39] and its consequences. However, to date, only 1 open-label randomized trial (with low statistical power) has tested the effect of 12-week oral CBD on the quality of life in PWH [40]. No significant treatment effect was found.

CBD is therefore likely to be well accepted by PWH and is expected to have a positive impact on both physical and psychological determinants of their HRQoL. Poor-to-modest quality evidence points to the beneficial effects of e-health or social and behavioral interventions for improving the quality of life of PWH [41, 42]. Combined aerobic and resistance exercise interventions have also shown benefits on several HRQoL domains for PWH [43]. Therefore, CBD can be considered as a candidate treatment on its own or in combination with other interventions.

We compiled data from a double-blind, randomized, placebo-controlled trial (designed for another primary objective) to test the hypothesis that PWH with an undetectable HIV viral load receiving medical full-spectrum CBD oil (1 mg/kg, twice per day for 12 weeks) would see an improvement in HRQoL.

## METHODS

### Participants

The double-blind, randomized, placebo-controlled trial (ClinicalTrials.gov Identifier: NCT05306249, registered on 04/01/2022) was conducted in France in 2022. Its primary objective was to assess the effect of CBD on autophagy-related gene expression in PWH. Assessing the effect of medical full-spectrum CBD on HRQoL (ie, the work described here) was a secondary objective.

Trial recruitment started in May 2022 and ended in October 2022. People with HIV-1, followed in the Department of Infectious and Tropical Diseases of the Regional Hospital Center of Orléans, were invited to participate during a follow-up visit. Those who agreed to participate and who met all the clinical trial's inclusion and exclusion criteria were invited back 3 days after the visit to provide written informed consent and to be randomized.

Inclusion criteria were as follows: aged 18 years or older at the moment the participant provided signed informed consent, having HIV-1 without HIV-2 co-infection, documented evidence of HIV plasma RNA assays <50 copies/mL during the 3 years preceding trial inclusion (occasional blips were tolerated), HIV-1 plasma RNA assay <50 copies/mL at inclusion, uninterrupted antiretroviral therapy during the 3 months before inclusion, receiving active contraception (for women of childbearing age), affiliated with French universal healthcare (“sécurité sociale,” which implied the reimbursement of usual health management costs), and able to provide informed written consent.

Exclusion criteria were as follows: pregnancy, breastfeeding (or planning to become pregnant or breastfeed during the trial), any sign of clinical stage III disease as classified by the Centers for Diseases Control and Prevention, taking an antiretroviral therapy containing a strong cytochrome P3A4 inhibitor (ritonavir or cobicistat) or efavirenz, receiving long-term nonsteroidal anti-inflammatory drugs or corticosteroids, taking recreational drugs including cannabis in the previous 6 months, personal history of psychotic disorder, history of severe cerebrovascular disease (ischemic or hemorrhagic stroke), renal failure (defined by a creatinine clearance <60 mL/min calculated according to “modification of diet in renal disease” equation), severe hepatic impairment (Child Pugh class C), unstable liver disease (defined by the presence of ascites, encephalopathy, coagulopathy, hypoalbuminemia, esophageal or gastric varices or persistent jaundice), cirrhosis, known biliary abnormality, disease or history of severe cardiovascular or cerebrovascular disorders, anticipated need for hepatitis C virus treatment during the randomization phase of the trial, current or past allergy or intolerance to CBD or to the terpenes contained in the trial product, active malignant tumor, presenting—in the opinion of the investigator—a significant risk of suicide, any preexisting physical or mental condition that could have interfered with the patient's ability to comply with CBD/placebo administration schedules or protocol evaluations, or that could have compromised patient safety, any condition that was likely to interfere with the absorption, distribution, metabolism, or elimination of trial drugs that could have prevented the patient from taking oral therapy, being deprived of liberty or institutionalized, being under tutorship, curatorship, or safeguard of justice, participating in another clinical trial evaluating a treatment, and finally, having a chronic inflammatory disease capable of altering the baseline level of cytokines (chronic inflammatory rheumatism, inflammatory bowel diseases, and immune-mediated inflammatory diseases).

### Sample Size

The trial's calculated sample size was based on the primary objective of assessing a difference in autophagy-related gene expression in peripheral blood mononuclear cell with an alpha risk of 5%, a beta risk of 10%, and a standard deviation based on a previous study [44].

### Trial Design and Study Treatment

CBD was the investigational medicinal product (IMP). Participants were randomly assigned, with a 1:1 allocation ratio, to either receive a double-blind 12-week course of the IMP (1 mg/kg, twice per day) (CBD group hereafter) or a placebo, plus a 4-week follow-up. Participants came to their recruitment center at 4-week intervals for measurements (W0, W4, W8, W12, W16).

The 12-week treatment period was initially based on unpublished preliminary data regarding the effects of CBD as a dietary supplement on the expression of several autophagy-related genes (the primary objective of the IMP trial). This 12-week period was also deemed sufficient to observe changes in HRQoL (or symptoms expected to impact it) according to studies conducted in other contexts [45–47]. The duration of the washout period was chosen to ensure the clearance of CBD from participants’ plasma [48].

The IMP comprised an orally administered oil formulation (CBD 50 mg/mL; CBD 50 LGP CLASSIC; Little Green Pharma, Perth, WA, Australia). The placebo was an orally administered formulation consisting of medium chain triglyceride oil which resembled the IMP in color, texture, and smell. Both the IMP and placebo appeared as a thick liquid in a dark 50-mL glass bottle with appropriate clinical trial labels attached. The treatment was the only difference in intervention between both groups.

The study was designed and implemented in accordance with the Declaration of Helsinki, and the protocol was approved by the Ethics Committee Ouest 6 (#CPP 1431 ME1, 29/11/2021- EudraCT 2020-005851).

### Data Collection

At W0 (treatment initiation), urine samples were taken to test for pregnancy and cannabis or CBD use. Urinary pregnancy tests were also performed at W4, W8, and W12. Blood samples were taken at all treatment and follow-up visits. At W0, W4, W12, and W16, HIV plasmatic viral load, CD4 and CD8 cell counts were assessed. The date of HIV diagnosis and the date of the first persistent undetectable viral load (defined as <50 copies/mL) were retrieved from each participant's computerized medical records.

At W0, W12, and W16, participants self-administered the 36-Item Short Form Health Survey questionnaire (SF-36) [49–51]. SF-36 is a generic instrument commonly used in PWH to assess HRQoL [52].

Data on adverse effects and their severity were collected throughout the study.

### Study Outcomes

The SF-36 contains 36 items measuring 8 domains of HRQoL: physical functioning, role limitations because of physical health problems, bodily pain, general health perceptions, vitality, social functioning, role limitations because of emotional problems, and mental health. All but 1 of the 36 items (item 2) are used to score these 8 domains, which can then be aggregated in 2 summary measures, called the physical and mental component summaries (PCS and MCS, respectively) [53, 54]. Therefore, a total of 10 HRQoL scores can be derived from the questionnaire. The SF-36 subscale scores and the PCS and MCS yield high levels of reliability and validity [51, 53]. The primary outcomes of the present study were the PCS and MCS. The secondary outcomes were the 8 subscales.

All SF-36 items were recoded on a 0 to 100 range so that the lowest and highest possible scores were 0 and 100, respectively, with higher scores indicating better HRQoL. The average of all the values for items in the same scale provided the overall score for each of the 8 domains. The PCS and MCS scores were then derived from these 8 domain scores using a 3-step process to ensure a mean score of 50 and a standard deviation of 10 [51, 53, 54].

Two time points were considered for the outcomes: W12 (end of treatment) and W16 (end of follow-up).

Because adverse effects were likely to impact HRQoL, the number of total and treatment-related adverse effects were compared between both treatment groups (ie, CBD and placebo).

### Explanatory Variables

Age and sex were tested as potential explanatory variables. To account for the impact of HIV on HRQoL, we also included the CD4/CD8 ratio [55] and the time since the first persistent undetectable viral load as adjustment variables.

### Statistical Analyses

Analyses were performed using a modified intent-to-treat approach. The modified intent-to treat population was defined as all patients who completed the W0 HRQoL assessment. We tested the randomness of missing values using a generalized estimating equation for probit model, with age, sex, CD4/CD8 ratio, and time since the first persistent undetectable viral load as explanatory variables.

Characteristics of the study population at W0 were described and compared between the 2 treatment groups (chi-square and Wilcoxon-Mann-Whitney tests for categorical and continuous variables, respectively). The Wilcoxon rank-sum test was used to compare before-after changes (W0–W12 and W0–W16) in outcomes in both groups.

Generalized estimating equation models were used to test the effect, if any, of the treatment group on SF-36 scores. Outcomes at W12 and W16 were modelled as a function of W0 value, follow-up visit (ie, W16 vs W12), treatment group, and visit × treatment group interaction with adjustments for age, sex, CD4/CD8 ratio, and time since the first persistent undetectable viral load. The identity function was applied as the link function, and the exchangeable correlation structure was applied.

The number of reported adverse effects in both treatment groups was compared using the Wilcoxon-Mann-Whitney test and binomial tests.

All analyses were performed with Stata version 17.0 for Windows (StataCorp LP, College Station, TX).

## RESULTS

### Study Sample Characteristics

**Figure 1.**
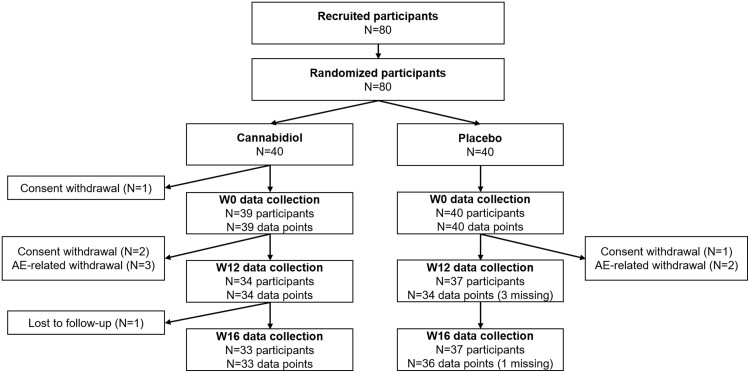
Flow chart of the study sample. AE, adverse effect; W, week.

Figure 1 is a flowchart of the study sample. A total of 80 participants were recruited in the trial. Of these, 79 were included in the present modified intent-to-treat analyses.

Table 1 provides participants’ characteristics at baseline (W0) according to treatment group. A majority of the sample were males (69.6%), median age was 56.4 years, median time since HIV infection was 18.9 years, and median time since their first persistent undetectable HIV viral load was 12.2 years.

**Table 1.** Study Sample Baseline Characteristics (N = 79)

…	Whole Study Sample	Cannabidiol (N = 39)	Placebo (N = 40)	*P* Value^a^
	N (%)	N (%)	N (%)	
**Sex**	…	…	…	.678
Male	55 (69.6)	28 (71.8)	27 (67.5)	…
Female	24 (30.4)	11 (28.2)	13 (32.5)	…
**Age (y, median [IQR])**	54.6 [48.5–65.4]	53.4 [48.0–60.3]	56.5 [49.3–68.2]	.192
**Time since HIV infection (y, median [IQR])**	18.9 [11.7–24.8]	19.1 [12.9–24.2]	16.8 [10.8–26.7]	.746
**Time since the first persistent undetectable HIV viral load (y, median [IQR])**	12.2 [8.1–17.3]	11.9 [9.2–17.3]	12.2 [7.7–16.8]	.610
**CD4 cell count (cells/µL, median [IQR])**	753 [563–930]	763 [596–933]	665.5 [555.5–923.5]	.436
**CD8 cell count (cells/µL, median [IQR])**	696 [469–924]	602 [455–878]	732 [488.5–942.3]	.486
**CD4/CD8 cell count ratio (median, [IQR])**	1.1 [0.7–1.6]	1.2 [0.7–1.6]	1.1 [0.7–1.6]	.314
**Antiretroviral treatments**	…	…	…	…
Nucleoside reverse transcriptase inhibitors	46 (58.2)	20 (43.5)	19 (57.6)	.216
Nonnucleoside reverse transcriptase inhibitors	43 (54.4)	24 (55.8)	15 (41.7)	.210
Integrase inhibitors	53 (67.1)	29 (54.7)	10 (38.5)	.174
**HIV viral load**	…	…	…	.201
<20 copies/mL	74 (93.7)	35 (89.7)	39 (97.5)	…
≥20 copies/mL	5 (6.3)	4 (10.3)	1 (2.5)	…

^a^Wilcoxon-Mann-Whitney for continuous variables, chi-square, or Fisher exact test for categorical ones.

Abbreviation: IQR, interquartile range.

**Figure 2.**
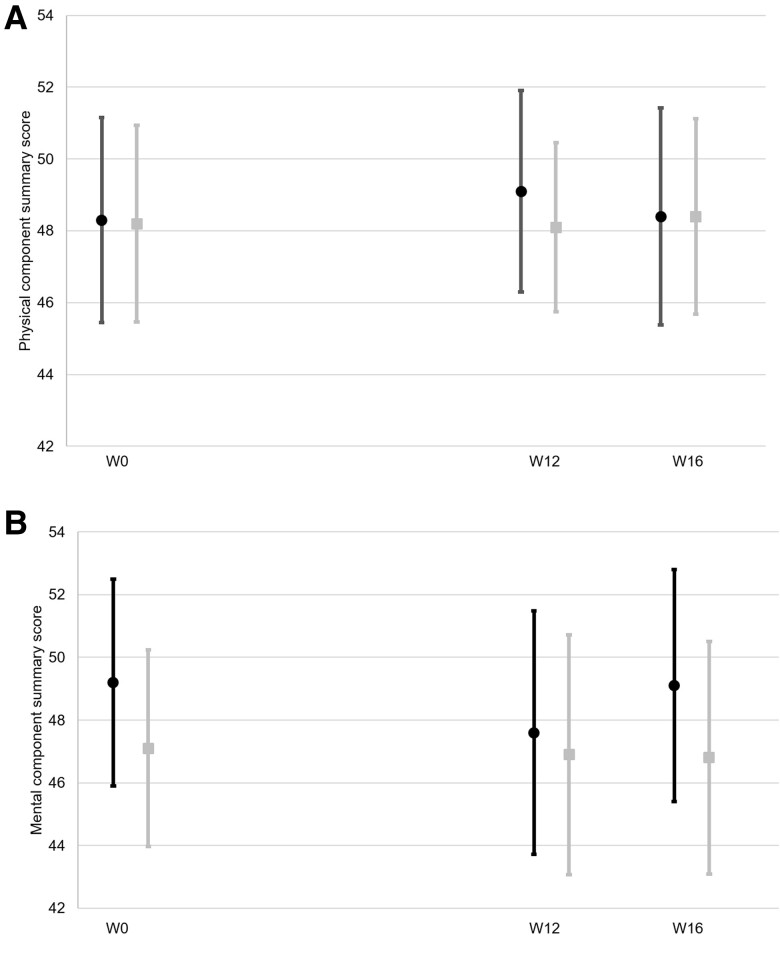
Evolution of SF-36 component summary scores according to treatment group. (*A*) Physical component summary, (*B*) Mental component summary; 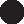, cannabidiol group; 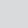 placebo group. Means and 95% confidence intervals are provided. W, week.

Supplementary Table 1 provides participants’ HRQoL scores at each visit according to treatment group. At W0, the median [interquartile range] PSC and MSC scores were, respectively, 49.1 [42.8–55.0] and 49.6 [43.1–55.8] in the whole-study sample. There was no difference for any score between both treatment groups at any visit. Similarly, there was no intra-group difference between the W0, W12, or W16 scores (Figure 2).

The proportion of detectable plasmatic viral load did not differ according to treatment group at any time point (Supplementary Table 2).

There was no missing data at W0. At W12, 10 participants had no data for the 10 scores (5 in each treatment group). At W16, 10 participants had no data for the 10 scores (6 in the CBD group, 4 in the placebo group). According to probit model, data were missing completely at random (data not shown).

### Treatment Effect on Health-related Quality of Life

In the models (adjusted for age, sex, CD4/CD8 ratio, time since the first persistent undetectable viral load, the follow-up visit, treatment group, and visit × treatment group interaction), higher W0 scores were associated with higher follow-up scores for all 10 HRQoL scores. There was no significant main effect of visit for any of the 10 scores. The only significant main effect of treatment group was observed for the physical functioning score, with a higher score for participants in the CBD group (regression coefficient [95% confidence interval] of 7.72 [0.55–14.89], *P* = .035) (Table 2). Post hoc analyses revealed a statistically significant difference for the physical functioning score at W12 between both treatment groups, but not at W16 (*P* = .168, data not shown).

**Table 2.** Treatment Effect on Health-related Quality of Life Scores (Generalized Estimating Equation Models, N = 79)

…	Regression Coefficient	*P* Value
	[95% CI]	
**Physical Component Summary**	**…**	…
W16 visit (ref. = W12 visit)	0.58 [−1.32 to 2.48]	.551
W0 value effect	0.53 [0.38–0.67]	…
Cannabidiol (ref. = placebo)	1.47 [−1.48 to 4.41]	.329
W16 visit × cannabidiol	−1.32 [−4.04 to 1.41]	.343
**Mental Component Summary**	**…**	…
W16 visit (ref. = W12 visit)	0.19 [−2.47 to 2.84]	.891
W0 value effect	0.67 [0.47–0.87]	<.001
Cannabidiol (ref. = placebo)	−0.19 [−4.42 to 4.04]	.930
W16 visit × cannabidiol	1.40 [−2.39 to 5.19]	.470
**Physical Functioning**	…	…
W16 visit (ref. = W12 visit)	1.99 [−1.64 to 5.62]	.283
W0 value effect	0.61 [0.44–0.78]	<.001
Cannabidiol (ref. = placebo)	7.72 [0.55–14.89]	.035
W16 visit × cannabidiol	−2.64 [−7.84 to 2.55]	.318
**Role Physical**	…	…
W16 visit (ref. = W12 visit)	−2.88 [−13.36 to 7.60]	.591
W0 value effect	0.30 [0.14–0.47]	<.001
Cannabidiol (ref. = placebo)	−7.66 [−20.78 to 5.46]	.253
W16 visit × cannabidiol	6.57 [−8.45 to 21.58]	.391
**Bodily Pain**	…	…
W16 visit (ref. = W12 visit)	5.41 [−0.16 to 10.98]	.057
W0 value effect	0.50 [0.33–0.67]	<.001
Cannabidiol (ref. = placebo)	7.55 [−1.75 to 16.84]	.111
W16 visit × cannabidiol	−9.00 [−16.97 to −1.04]	.027
**General Health**	…	…
W16 visit (ref. = W12 visit)	−0.074 [−4.07 to 3.93]	.971
W0 value effect	0.76 [0.61–0.91]	<.001
Cannabidiol (ref.=placebo)	1.09 [−5.57 to 7.56]	.747
W16 visit × cannabidiol	0.39 [−5.33 to 6.11]	.894
**Vitality**	…	…
W16 visit (ref. = W12 visit)	0.76 [−4.03 to 5.55]	.755
W0 value effect	0.65 [0.47–0.83]	<.001
Cannabidiol (ref. = placebo)	4.43 [−2.30 to 11.16]	.197
W16 visit × cannabidiol	−2.75 [−9.60 to 4.11]	.432
**Social Functioning**	…	…
W16 visit (ref. = W12 visit)	1.51 [−4.52 to 7.54]	.623
W0 value effect	0.54 [0.34–0.74]	<.001
Cannabidiol (ref. = placebo)	−0.81 [−10.16 to 8.53]	.865
W16 visit × cannabidiol	1.32 [−7.31 to 9.94]	.765
**Role Emotional**	…	…
W16 visit (ref. = W12 visit)	1.97 [−11.41 to 7.47]	.682
W0 value effect	0.43 [0.26–0.61]	<.001
Cannabidiol (ref. = placebo)	−5.62 [−18.49 to 7.24]	.391
W16 visit × cannabidiol	10.26 [−3.26 to 23.77]	.137
**Mental Health**	…	…
W16 visit (ref. = W12 visit)	1.74 [−2.88 to 6.36]	.461
W0 value effect	0.80 [0.61–0.98]	<.001
Cannabidiol (ref. = placebo)	4.11 [−2.83 to 11.06]	.246
W16 visit × cannabidiol	−2.27 [−8.88 to 4.34]	.501

Models were adjusted for age, sex, CD4/CD8 cell count ratio, and time since the first persistent undetectable viral load.

CI, confidence interval; ref., reference; W, week.

The only significant visit × treatment group interaction effect was observed for the bodily pain score (−9.00 [−16.97 to −1.04], *P* = .027, Table 2). Post hoc analyses revealed that this score was 3.59 points lower at W16 (compared with W12) for participants in the CBD group (*P* = .216, data not shown). This decrease was not significant.

### Adverse Effects

Table 3 provides the number of adverse effects reported in each treatment group. Of the total 109 adverse effects, 56 were reported by participants in the CBD group: the number of treatment-related adverse effects did not differ between the 2 groups. All adverse effects were of light-to-moderate intensity, with the exception of 1 severe adverse effect, unrelated to the treatment. The most common treatment-related adverse effects were “discomfort in the throat” (n = 5), “fatigue/drowsiness” (n = 2), “impairment of sleep quality” (n = 2), and “nausea/disgust” (n = 2). The most common treatment-unrelated adverse effects were “fatigue/drowsiness” (n = 10), “COVID-19” (n = 6), “headache” (n = 6), and “gastrointestinal disorders” (n = 5). It should be noticed that no up-titration schedule (ie, slowly increasing the IMP dose by small amounts over days) had been done during this trial, which is the now recommendations for CBD medication.

**Table 3.** Adverse Effects According to Treatment Group

…	Total (N = 79)	Cannabidiol (N = 39)	Placebo (N = 40)	*P* Value
Total number of adverse effects	109	56	53	.740^a^
Number of participants experiencing at least 1 adverse effect	56	28	28	1.00^b^
Total number of treatment-related adverse effects	15	7	8	.616^a^
Adverse effects-related withdrawal	5	3	2	.625^a^

^a^Wilcoxon-Mann-Whitney test.

^b^Binomial test.

## DISCUSSION

To our knowledge, the double-blind, randomized, controlled trial we conducted is the first to assess the impact of medical full spectrum CBD on HRQoL among PWH on long-term antiretroviral therapy. We found no effect of CBD (1 mg/kg, twice per day for 12 weeks) intake on physical and mental component summary scores. However, we found that CBD intake was associated with a higher physical functioning score at W12 only. No significant main effect of CBD intake on the other HRQoL subscale scores was observed.

This limited beneficial impact of CBD on PWH's HRQoL is consistent with a recent open-label randomized trial in Canada (oral capsules consisted of 200–800 mg purified CBD in oil per day, n = 5), which found no significant effect of CBD on HRQoL [40].

A small number of controlled trials have assessed the impact of CBD on HRQoL for various health conditions other than HIV. For example, in patients with advanced cancer receiving palliative care, synthetic purified CBD oil (median dose of 400 mg CBD per day) for 28 days had no impact on quality of life [56]. Furthermore, in patients with ulcerative colitis, CBD-rich botanical extract (mean daily dose of 300 mg CBD) improved some ulcerative colitis–specific measures of quality of life more than a placebo [45]. Elsewhere, in patients with Parkinson's disease, 6 weeks of CBD-enriched cannabis product (15.6 mg CBD per day, associated with 0.61 mg of tetrahydrocannabinol per day) did not improve quality of life compared to a placebo [57]. Another study in patients with Parkinson's disease found that CBD 300 mg per day for 6 weeks improved the total Parkinson's Disease Questionnaire-39 score but not the Unified Parkinson's Disease Rating Scale total score; instead a 75 mg per day dose was ineffective [46]. Finally, a randomized, double-blind, placebo-controlled trial in patients with functional dyspepsia found no effect of pharmaceutical-grade CBD (20 mg/kg per day for 4 weeks) on quality of life [58]. Recently, from a case series of 3148 patients with various conditions who were taking cannabis for medical purposes, Arkell et al. found that for CBD-dominant products over 15 follow-up consultations, the SF-36 domains of general health, physical functioning, role-physical, mental health, and role-emotional, all showed improvements in univariate analyses [59]. However, after adjustment, these results were no longer significant. These various findings suggest that there is limited evidence for improved HRQoL following CBD administration, even in contexts where participants received higher CBD doses than the one we used in our trial (1 mg/kg, twice per day).

PWH commonly use cannabis to manage anxiety, stress, pain, and sleep disorders [17, 18], all of which are known determinants of impaired HRQoL. However, there is no evidence that CBD is effective in reducing any of those symptoms. Regarding anxiety, 2 recent randomized controlled trials, providing up to 800 mg per day, found no benefit of CBD [60, 61]. To date, randomized trials for pain have also failed to find any significant beneficial effect of CBD [62–67]. The same is true for sleep disorders [28], with the exception of 1 study that reported improved sleep quality with self-titrated CBD for 1 week in patients with chronic pain [68]. Finally, we found no data from randomized trials documenting the effects of CBD on depression [69]. This highlights the small number of randomized controlled trials on the effect of CBD (without tetrahydrocannabinol) for these various symptoms in isolation, and the total absence of double blinded studies on PWH. Furthermore, the lack of evidence of an effect of CBD on anxiety, stress, pain, and sleep disorders reflects the absence of a CBD impact on the PCS and MCS scores we found.

It is important to emphasize the beneficial effect of CBD we found on the physical functioning HRQoL domain. Specifically, when compared to the placebo, we found an improvement of 7.2 points at W12 but not at W16. This improvement may be considered to be clinically significant (according to the 3- to 5-point change identified by Samsa et al. [70]). Our results suggest that full-spectrum CBD may be of interest to PWH with impaired physical functioning, either as a standalone treatment or in combination with other interventions that have shown effects in this domain [42, 43]. The absence of any effect at W16 (end of the washout period) may be related to the transient nature of the CBD effect. The worsening of bodily pain between W12 and W16 in the CBD group we found may be related to a previous improvement between W0 and W12 that we failed to significantly identify.

The limited positive beneficial effects of CBD on HRQoL may partly stem from the stringent inclusion criteria applied. Indeed, it has been shown that HRQoL is inversely related to HIV viral load [71, 72]. By only including PWH with an undetectable viral load, we may have created a cohort of baseline participants with an already relatively high HRQoL, which meant there was limited place for improvement following administration of the IMP. This is illustrated by high SF-36 scores at baseline (PCS and MCS scores were close to the standardized mean of 50 [51]) compared to other studies in Europe [73, 74]. The same mechanism (ie, high initial HRQoL limiting level of improvement) is possible given that individuals with liver and/or kidney disease, cardiovascular or cerebrovascular disorders, chronic inflammatory disease, and mental conditions, were all excluded.

The 2 mg/kg daily dosage (equivalent to approximately 8 drops of a commercial 30% CBD oil for a 60-kg individual) may have been too low to detect clinical effects. Finally, because HRQoL was a secondary outcome of the trial, the sample may also have been too small to highlight more significant changes in HRQoL. Moreover, the relatively small sample size prevented us from exploring the interaction between the inflammatory status of participants and the treatment, which would have been valuable to understand the potential immunomodulatory effects of full-spectrum CBD. CBD oral bioavailability is low and likely to vary between individuals [75, 76]. Some of the effects of CBD may be modulated by changes in gut microbiota [39, 77]. Future studies should monitor CBD levels in blood as well as microbiota composition as potential mediators of the effects of CBD on HRQoL. Despite the limited positive impact of full spectrum CBD we evidenced, the absence of HRQoL deterioration in our study confirms the good tolerability of CBD in healthy, effectively treated (ie, undetectable HIV plasma viral load) PWH [40].

Our study has several strengths. First, to our knowledge it is the first randomized, double blind, controlled trial to test the effect of full-spectrum CBD on HRQoL in PWH on long-term antiretroviral therapy. Second, its double-blind design prevented the placebo effect frequently observed in cannabinoid-based trials [78]. Although our stringent inclusion criteria may have limited external validity, the homogeneity of our study population ensured internal validity. We cannot discard that our small sample size prevented us from detecting existing changes in HRQoL. However, we can expect that the relatively long duration of the trial would partly offset this limitation. Finally, it is possible that the SF-36 scale does not assess HIV-specific drivers of HRQoL. However, as these drivers (which are included in some HIV-specific HRQoL scales, for example HIV-related stigma and financial insecurity [52]) were not supposed to be impacted by the trial's IMP (ie, CBD), we believe that the SF-36 scale was a suitable choice for our study.

To conclude, twice-daily full-spectrum CBD oil at 1 mg/kg had no major effect on HRQoL in PWH with long-term undetectable HIV viral load. Large-size randomized controlled trials that include PWH with lower baseline HRQoL are needed to confirm this result.

## Supplementary Data

Supplementary materials are available at *Open Forum Infectious Diseases* online. Consisting of data provided by the authors to benefit the reader, the posted materials are not copyedited and are the sole responsibility of the authors, so questions or comments should be addressed to the corresponding author.

## Supplementary Material

ofae492_Supplementary_Data

## Notes

***Acknowledgments.*** We thank all the study participants and hospital staff who participated in the trial. Our thanks to Jude Sweeney (Milan) for English revision and copyediting.

***Financial support*.** The trial received financial support from SynCHROnie and Région Centre-Val de Loire (TRANSINFLA). Little Green Pharma Pty Ltd. and Intsel Chimos provided the investigational medicinal product (ie, CBD) and the placebo at no financial cost for the purposes of this research. The funders had no role in the writing of this manuscript or in the decision to publish it.

***Authors’ contributions.*** T.B. designed the study and wrote the original draft. C.C. collected data and revised the draft. A.M. performed statistical analyses and revised the draft. P.C. designed the study and revised the draft. H.K. provide treatment and revised the draft. B.D. collected data. L.H. recruited participant and collected data. L.M. conceptualized and designed the trial and revised the draft. T.P. conceptualized and designed the trial, recruited participants, and revised the draft.

***Data availability.*** The datasets generated and/or analyzed for the current study are available from the corresponding author on reasonable request.

***Patient Consent Statement.*** The patient's written consent was obtained. The study was designed and implemented in accordance with the Declaration of Helsinki, and the protocol was approved by the Ethics Committee Ouest 6 (#CPP 1431 ME1, 29/11/2021).
