# Supplementary material for: Limited Impact of Cannabidiol on Health-related Quality of Life of People With Long-term Controlled HIV: A Double-blind, Randomized, Controlled Trial
Source: Open Forum Infect Dis. 2024 Aug 27;11(9):ofae492. doi: 10.1093/ofid/ofae492 (PMC11409882; doi:10.1093/ofid/ofae492)
Supplement: ofae492_Supplementary_Data [file ofae492_supplementary_data.zip › BARRE_OFID_Supplementary_Table1.docx]

**Supplementary Table 1. Health-related quality of life scores according to treatment group**

|  | **Cannabidiol** | | | | | **Placebo** | | | | |
| --- | --- | --- | --- | --- | --- | --- | --- | --- | --- | --- |
| Median [IQR] | **W0 (N=39)** | **W12 (N=34)** | **W16 (N=33)** | **p-value (W0 vs. W12)^1^** | **p-value (W0 vs. W16)^1^** | **W0 (N=40)** | **W12 (N=34)** | **W16 (N=36)** | **p-value (W0 vs. W12)^1^** | **p-value (W0 vs. W16)^1^** |
| **Physical Component Summary** | 51.5 [44.0-54.7] | 51.5 [45.4-54.5] | 51.2 [43.1-55.0] | 0.101 | 0.458 | 49.0 [40.7-56.2] | 48.1 [42.9-54.7] | 49.9 [42.9-55.6] | 0.612 | 0.822 |
| **Mental Component Summary** | 51.0 [44.1-57.1] | 52.1 [44.3-55.6] | 51.7 [45.6-57.2] | 0.489 | 0.8511 | 48.5[42.1-54.1] | 48.8 [43.9-54.2] | 48.9[40.0-55.9] | 0.768 | 0.414 |
| **Physical Functioning** | 90  [80-100] | 95  [80-100] | 95 [78-100] | 0.194 | 0.421 | 90 [75-100] | 90 [70-100] | 90 [71-100] | 0.209 | 0.379 |
| **Role Physical** | 100  [75-100] | 100 [50-100] | 100 [75-100] | 0.941 | 0.456 | 100 [75-100] | 100 [75-100] | 100 [75-100] | 0.645 | 0.718 |
| **Bodily Pain** | 80  [45-100] | 90 [67-100] | 80 [45-100] | 0.174 | 0.957 | 73.8 [48.1-100] | 77.5[57.5-90] | 73.8 [58.1-100] | 0.669 | 0.214 |
| **General Health** | 70 [55-75] | 68 [55-80] | 65 [53-85] | 0.986 | 0.767 | 68 [45-79] | 65 [45-85] | 63 [46-75] | 0.586 | 0.690 |
| **Vitality** | 60 [45-65] | 60 [45-66] | 50 [45-70] | 0.904 | 0.725 | 64 [52-79] | 60 [52-80] | 64 [56-82] | 0.542 | 0.806 |
| **Social Functioning** | 87.5 [75-100] | 87.5 [59.4-100] | 87.5 [68.8-100] | 0.557 | 0.581 | 81.3 [53.1-100] | 87.5 [62.5-100] | 87.5 [62.5-100] | 0.319 | 0.204 |
| **Role Emotional** | 100  [67-100] | 100  [67-100 | 100  [100-100 | 0.445 | 0.297 | 100  [67-100] | 100  [100-100 | 100  [67-100 | 0.545 | 0.706 |
| **Mental Health** | 72 [52-84] | 76 [80-81] | 68 [52-84] | 0.904 | 0.725 | 64  [52-79] | 60 [52-80] | 64 [56-82] | 0.542 | 0.806 |

IQR, interquartile range.

^1^ Wilcoxon signed rank test.
