# Supplementary material for: Limited Impact of Cannabidiol on Health-related Quality of Life of People With Long-term Controlled HIV: A Double-blind, Randomized, Controlled Trial
Source: Open Forum Infect Dis. 2024 Aug 27;11(9):ofae492. doi: 10.1093/ofid/ofae492 (PMC11409882; doi:10.1093/ofid/ofae492)
Supplement: ofae492_Supplementary_Data [file ofae492_supplementary_data.zip › BARRE_OFID_Supplementary_Table2.docx]

**Supplementary Table 2. Proportion of detectable viral load according to treatment group**

|  | Cannabidiol | | | Placebo | | | p-value^1^ | | |
| --- | --- | --- | --- | --- | --- | --- | --- | --- | --- |
|  | W0 | W12 | W16 | W0 | W12 | W16 | W0 | W12 | W16 |
| **HIV viral load** |  |  |  |  |  |  | 0.201 | 1.00 | 1.00 |
| ≥ 20 copies/mL | 4 (10.3%) | 1 (2.9%) | 2 (6.1%) | 1 (2.5%) | 1 (2.7%) | 2 (5.4%) |  |  |  |
| <20 copies/mL | 35  (89.7%) | 33 (97.1%) | 31 (93.9%) | 39 (97.5%) | 36 (97.3%) | 35 (94.6%) |  |  |  |

**^1^** Fisher exact test
